# Fatal Flea-Borne Typhus in Texas: A Retrospective Case Series, 1985–2015

**DOI:** 10.4269/ajtmh.16-0465

**Published:** 2017-05-03

**Authors:** Emily G. Pieracci, Nicole Evert, Naomi A. Drexler, Bonny Mayes, Inger Vilcins, Philip Huang, Jill Campbell, Casey Barton Behravesh, Christopher D. Paddock

**Affiliations:** 1 Rickettsial Zoonoses Branch, Division of Vector-Borne Diseases, National Center for Emerging and Zoonotic Infectious Diseases, Centers for Disease Control and Prevention, Atlanta, Georgia; 2 Epidemic Intelligence Service, Center for Surveillance, Epidemiology and Laboratory Services, Centers for Disease Control and Prevention, Atlanta, Georgia; 3 Texas Department of State Health Services, Austin, Texas; 4 Austin/Travis County Health and Human Services Department, Austin, Texas

## Abstract

Flea-borne (murine) typhus is a global rickettsiosis caused by *Rickettsia typhi*. Although flea-borne typhus is no longer nationally notifiable, cases are reported for surveillance purposes in a few U.S. states. The infection is typically self-limiting, but may be severe or life-threatening in some patients. We performed a retrospective review of confirmed or probable cases of fatal flea-borne typhus reported to the Texas Department of State Health Services during 1985–2015. When available, medical charts were also examined. Eleven cases of fatal flea-borne typhus were identified. The median patient age was 62 years (range, 36–84 years) and 8 (73%) were male. Patients presented most commonly with fever (100%), nausea and vomiting (55%), and rash (55%). Respiratory (55%) and neurologic (45%) manifestations were also identified frequently. Laboratory abnormalities included thrombocytopenia (82%) and elevated hepatic transaminases (63%). Flea or animal contact before illness onset was frequently reported (55%). The median time from hospitalization to administration of a tetracycline-class drug was 4 days (range, 0–5 days). The median time from symptom onset to death was 14 days (range, 1–34 days). Flea-borne typhus can be a life-threatening disease if not treated in a timely manner with appropriate tetracycline-class antibiotics. Flea-borne typhus should be considered in febrile patients with animal or flea exposure and respiratory or neurologic symptoms of unknown etiology.

## Introduction

Flea-borne typhus, also known as endemic or murine typhus, is a rickettsial zoonosis caused by *Rickettsia typhi* that occurs predominantly in warm, coastal areas of the world, including certain parts of the United States.^1^ During the early 1940s, approximately 2,000–5,000 cases were reported annually, but sanitation and vector control campaigns significantly reduced the number of flea-borne typhus cases in the United States. In 1995, flea-borne typhus was removed from the list of nationally notifiable diseases,^2^ making the contemporary prevalence of flea-borne typhus in the United States difficult to ascertain^3^,^4^; nonetheless, flea-borne typhus remains a reportable disease in 14 U.S. states (CA, HI, IL, IN, MI, MN, MS, NE, NH, OH, OR, PA, TX, and WA). The majority of U.S. cases occur in Texas, which reported 3,048 confirmed or probable cases of flea-borne typhus during 1985–2015 (Texas Department of State Health Services, unpublished data). Flea-borne typhus is often described as a relatively mild and self-limiting rickettsiosis; nonetheless, a spectrum of severe manifestations is also recognized,^5^–^10^ and the disease may be fatal in as many as 5% of patients for whom appropriate antibiotic therapy is delayed or not provided.^11^ The objective of this study was to describe the clinical and epidemiologic features associated with fatal flea-borne typhus cases in Texas.

## Methods

Flea-borne typhus cases are reported in Texas by using a standardized case report form that includes demographic information, various clinical characteristics, duration of illness, animal and arthropod exposure, travel history, and outdoor activities prior to illness onset. The data were reviewed for confirmed and probable fatal cases identified during 1985–2015. When available, medical charts were examined for supplemental information relating to comorbidities, clinical presentation, antibiotic therapy, laboratory results, and diagnosis at time of death. Diagnostic test results were generated by multiple commercial laboratories or by state or national reference centers. All personally identifiable information was removed from the case investigation forms and medical charts before review at the Centers for Disease Control and Prevention (CDC). This case series protocol was reviewed and determined to qualify as non-research and was exempt from further review according to the CDC's National Center for Emerging and Zoonotic Infectious Diseases institutional procedures.

A confirmed case was defined as a patient with a clinically compatible illness and 1) a 4-fold or greater rise in antibody titer to *R. typhi* antigens by using an indirect immunofluorescence antibody (IFA) assay in acute and convalescent phase serum specimens; or 2) a single reciprocal IFA titer ≥ 1,024; or 3) a positive polymerase chain reaction (PCR) assay to *R. typhi*; or 4) demonstration of typhus group *Rickettsia* sp. antigens by an immunohistochemical stain in tissue; or 5) isolation of *R. typhi* from clinical specimen. A probable case was defined as a patient with a clinically compatible illness and a reciprocal IgG or IgM IFA titer of ≥ 64.

## Results

**Figure 1.**
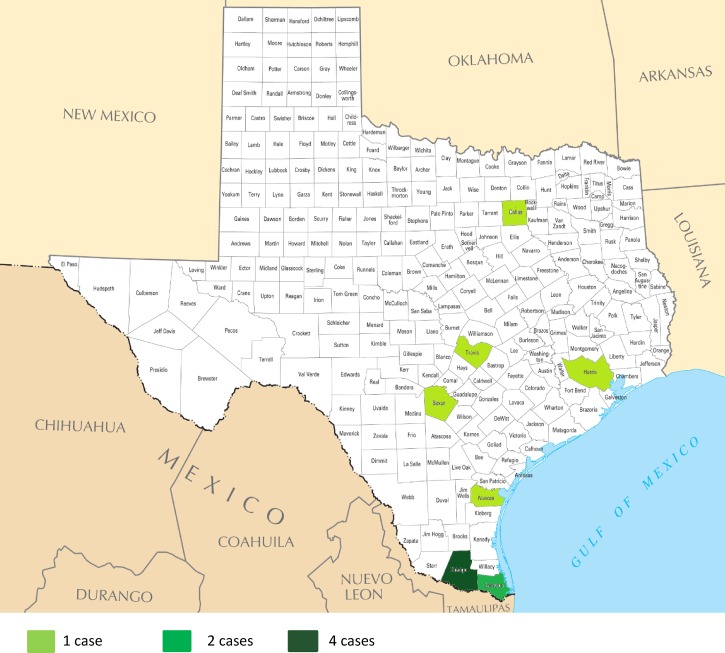
Texas counties with fatal flea-borne typhus by case count, 1985–2015.

**Figure 2.**
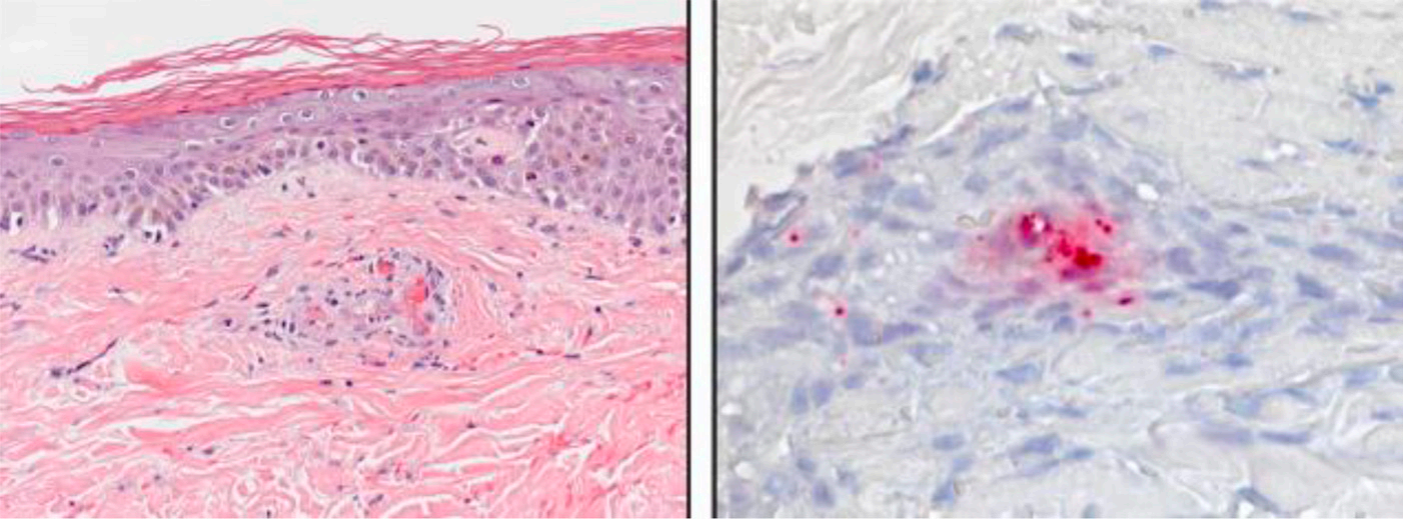
Histopathological and immunohistochemical appearance of a skin biopsy specimen from a patient with a fatal typhus group rickettsiosis.

Eleven confirmed or probable fatal cases of flea-borne typhus were reported in Texas during 1985–2015 (Figure 1

). The median age of the patients was 62 years (range, 36–84 years) and 9 (82%) were > 50 years of age. Eight (73%) patients were male and seven (64%) were of Hispanic ethnicity (Table 1). Six patients met the criteria for a confirmed case; four (67%) patients were diagnosed with single reciprocal IFA titers ≥ 1,024 (range, 1,024–4,096); one patient demonstrated a 4-fold rise in antibody titer; one patient had a skin biopsy specimen that stained positive for antigens of a typhus group *Rickettsia* sp. (Figure 2

) and a reciprocal IFA titer of ≥ 4,096 (Table 1). Five patients met the criteria for a probable case and were classified using a reciprocal serologic titer < 1,024 (range, 64–512). Of these, one had reciprocal IgG and IgM titers of 256 and 2,048, respectively, 5 days post-illness onset, and one had reciprocal IgG and IgM titers of 64 and 512, respectively, 9 days after illness onset (Table 1). No patients were diagnosed by PCR or culture isolation. Five (38%) patients were additionally tested by an IFA assay using antigens of *Rickettsia rickettsii* (the agent of Rocky Mountain spotted fever [RMSF]); 2(40%), 1(20%), and 2(40%) had reciprocal IgG antibody titers to this antigen at < 64, 128, and 512, respectively. Both patients with *R. rickettsii* titer of 512 had *R. typhi* titers ≥ 4,096. For the patients with reciprocal titers to both rickettsial antigens, the titer to *R. typhi* was consistently 4-fold or greater than the titer to *R. rickettsii*. Two additional patients were identified in 1999 and 2001. These patients each had reciprocal IgG antibody titers to *R. typhi* of 512; however, no additional case information was available on their clinical course of illness and they were excluded from further analysis.

Eight of 11 (73%) case report forms contained information on flea or animal contact in the 2 weeks before illness onset. Three (27%) patients reported fleas in the home, three (27%) patients had at least one dog, and four (36%) patients either owned a cat or cared for stray cats. Two (18%) patients reported an opossum living within or next to their home (Table 1). One (9%) patient reported hunting in south Texas before illness onset, but no specific animal species were named. Cases occurred year-round with no apparent seasonality (Table 1).

All patients were hospitalized. At hospital presentation, fever was reported for 11 (100%) patients. The median temperature was 101.9°F (range, 99–104.6°F). Thrombocytopenia (82%) and elevated hepatic transaminase levels (64%) were the most frequently reported laboratory abnormalities. Six (55%) patients reported one or more respiratory-related symptom (i.e., cough), or diagnosis (i.e., pneumonia, pulmonary edema, or acute respiratory distress syndrome). Rash was reported in 55% of patients, and was described as macular and maculopapular (50%) or petechial (50%), and was distributed across the palms and soles (33%), arms and legs (33%), and trunk (67%) (Table 2). Rash was frequently reported as a late finding, occurring > 7 days after initial illness onset.

The median time from symptom onset to hospitalization was 8 days (range, 1–21 days), and the median time from symptom onset to death was 14 days (range, 1–34 days). Four (36%) patients sought medical care during their first week of illness; two cases were diagnosed with urinary tract infections unrelated to *R. typhi* infection and prescribed non-tetracycline class antibiotics, one patient received an unknown antibiotic for an unspecified illness, and the fourth patient had no information available. Two (18%) patients received a sulfa-containing antibiotic during their illness. Seven (64%) patients received a tetracycline-class antibiotic (doxycycline or tetracycline) during hospitalization. The median number of days from hospital admission to initiation of a tetracycline-class antibiotic was 4 (range, 0–5 days). The median time from symptom onset to initiation of a tetracycline-class drug was 9 days (range, 4–17 days).

Additional data abstracted from medical charts were available for eight patients. Of these, neurologic complications were present at hospital admission in five (63%) patients and included meningitis, encephalitis, vertigo, dizziness, seizure, and coma. Two of the five patients were obtunded on presentation and never regained consciousness. At hospital presentation, two (25%) patients had acute kidney injury. Comorbidities were for five (63%) of these patients including three (37%) with a history of alcohol abuse, one (13%) with chronic renal disease, congestive heart failure, and Type 2 diabetes mellitus, and one (13%) with a preexisting seizure disorder.

## Discussion

To our knowledge, this is the largest series of patients with fatal flea-borne typhus. The case fatality rate (CFR) in Texas during this period (0.4%) was not significantly different from the CFR described for 200 cases of flea-borne typhus from Texas that occurred during 1980–1984^12^ (1%, *P* = 0.30). During the pre-antibiotic era, CFRs of flea-borne typhus varied regionally, but the overall CFR for 18,337 U.S. cases during 1940–1944 was 4.6%.^11^–^14^ Among the cases reviewed herein, a high proportion were male (73%) and over the age of 50 years (82%). More than 70% of the patients in this series who were treated with a tetracycline-class antibiotic did not receive this drug until the second week of illness, emphasizing the need for prompt administration of appropriate antimicrobial therapy to reduce fatal outcome.

Respiratory signs or symptoms were noted for more than half of the patients in this series. The frequency of respiratory manifestations have been recognized for many years; indeed, an early summary of flea-borne typhus in Texas noted, “few diseases present so much subjective respiratory symptoms with so little objective findings.”^15^ Previous literature reported 14–59% of flea-borne typhus patients had respiratory symptoms.^12^,^16^,^17^ Neurologic manifestations were also seen in more than half of the patients for whom additional data were available, and previous literature has described neurologic manifestations in 15–45% of patients.^17^,^18^ Our findings are consistent with previous literature and suggest that respiratory and neurologic complications from *R. typhi* infection may occur frequently in severe cases.^6^,^16^,^18^–^21^ The frequency of respiratory and neurologic manifestations identified in this series may have been confounded by the advanced stage of disease that patients presented at initial hospital presentation. Previous literature reports risk of complications increases with age. Therefore, older patients (> 50 years) may be at greater risk for poor outcomes.^20^,^22^,^23^

Of the seven patients with elevated hepatic transaminases, three (43%) had a history of alcohol abuse. Clinicians may have attributed elevated hepatic transaminases to complications from alcohol abuse; however, hepatic inflammation has also been documented with severe flea-borne typhus.^24^,^25^ Two patients in this case series were treated with sulfa-containing antibiotic after illness onset and before hospital admission. Multiple historical and contemporary case reports describe adverse outcomes in flea-borne typhus patients treated with sulfa-containing antibiotics.^11^,^18^,^26^ Similar observations have been identified in patients with other rickettsial diseases, particularly RMSF and *Ehrlichia chaffeensis* ehrlichiosis.^27^ Case–control studies are needed to better assess the potential contraindication of sulfa drugs in the treatment of patients with rickettsiosis, including flea-borne typhus. Glucose-6-phosphate dehydrogenase deficiency was not identified or assessed as a comorbidity in any of the patients in this series; nonetheless, this condition has been previously associated with life-threatening disease in patients with flea-borne typhus^28^ as well as other rickettsioses including RMSF^29^ and Mediterranean spotted fever (caused by *Rickettsia conorii*)^30^ and scrub typhus (caused by *Orientia tsutsugamushi*).^28^

Five cases of fatal flea-borne typhus have been reported previously in detail.^20^,^26^,^31^ These patients, from the United States and Spain, presented with similar demographic and clinical characteristics to the patients in our case series. Their median age was 56 years (range, 46–81 years), three (60%) were male, one (20%) reported contact with rodents, four (80%) reported fever, one (20%) had rash, two (40%) had thrombocytopenia, one (20%) developed respiratory manifestations, and one (20%) developed neurologic manifestations. The median time from illness onset to death was 13 days (range, 11–15 days). Importantly, two (40%) received a sulfa-containing antibiotic.

In previous outbreaks, as many as 70% of patients of flea-borne typhus have required hospitalization and as many as 30% of these patients require admission to an intensive care unit^32^; nonetheless, even in areas such as Texas where the disease is endemic, a correct diagnosis of flea-borne typhus is often delayed for 10 or more days.^33^ The signs and symptoms of even advanced disease may be nonspecific and can hamper a timely diagnosis. Delay in treatment with a tetracycline-class antibiotic is associated with poorer outcomes including death. Doxycycline is the drug of choice for treating rickettsiosis in patients of all ages.^34^ Flea-borne typhus should be considered in patients with a history of animal or flea exposure and a nonspecific febrile illness of > 3 days.^22^,^31^,^34^ Absence of rash should not exclude a presumptive diagnosis of flea-borne typhus, particularly as it often occurs late in the development of disease, as identified in this series. A history of recent exposure to fleas or animals can provide valuable clues into potential etiology of unknown illness; however, absence of known animal or flea exposures does not rule out flea-borne typhus in an endemic area, as patients may be unaware of these exposures.

This case series had several limitations inherent to studies that use data acquired predominantly or exclusively through passive surveillance, including incompleteness of information reported to the state health department, and inability to review medical charts for some of the patients. In contrast to many other rickettsioses endemic in the United States, typhus group rickettsioses are not nationally notifiable. In that context, there are no nationally recognized laboratory criteria to define confirmed or probable cases of typhus group rickettsioses. Because each of the fatal cases were from Texas, we applied the case definitions developed by the Texas Department of State Health Services. Nonetheless, a single antibody titer is not a universally accepted laboratory criterion for a confirmed case. Furthermore, because this was a retrospective study, the serological data were generated by multiple laboratories during a 30-year period and therefore lack consistency with respect to reagents, methods, and interpretation by laboratory personnel. Finally, this study lacked a control group to comparatively assess risk factors. Future case–control studies could help identify risk factors for severe and fatal outcomes in patients with flea-borne typhus.

Infection with *Rickettsia felis*, a typhus-like *Rickettsia*, has been documented in at least one patient in Texas.^35^ Because *R. typhi* and *R. felis* occur sympatrically in fleas in Texas, it is possible that *R. felis* may be responsible for some cases of flea-borne typhus^36^–^38^; however, vertebrates infected with *R. felis* characteristically produce antibodies that cross-react with antigens of spotted fever group rickettsiae rather than typhus group rickettsiae.^39^,^40^ Antibodies reactive with spotted fever group rickettsiae were detected in five of the patients in this series for whom these tests were performed, but each of these patients had corresponding typhus group titers that were at least 4-fold higher. Additionally, *R. typhi* typically causes more severe disease in humans than *R. felis*,^6^,^19^,^22^ and to our knowledge there are no reported fatal cases of human infection with *R. felis*. A previous study of Texas patients for whom a flea-borne rickettsiosis was suspected found evidence of infection with *R. typhi* in 22% versus infection with *R. felis* in only 2%, by using species-specific recombinant antigens, to suggest that infections with *R. typhi* are more prevalent in humans in Texas than are infections with *R. felis*.^41^ In this context, we believe each of the fatal cases described herein represented infection with *R. typhi* rather than *R. felis*. A confirmed human infection with *Rickettsia prowazekii*, another typhus group *Rickettsia* species, was described in 2001 that may have been acquired in south Texas^42^ and serological evidence of infection with *R. prowazekii* was confirmed by cross-adsorption assays in two of 19 homeless persons in Houston, TX, with antibodies reactive to typhus group rickettsiae.^43^ *Rickettsia prowazekii* has been detected in an *Amblyomma* tick collected in northern Mexico,^44^ and this species is also found in south Texas.^45^ In this context, it is also possible that some of the cases in this case series were caused by *R. prowazekii* rather than *R. typhi*.

Clinicians may want to consider older age, delay in treatment, and a history of alcohol abuse as potential risk factors for severe flea-borne typhus; however, case–control studies need to be conducted to identify risk factors for severe disease. Severity of disease may be mitigated by early diagnosis and initiation of appropriate tetracycline-class antibiotic therapy. Clinicians should be aware that flea-borne typhus is a potentially serious illness and should be considered in febrile patients with animal or flea exposure and respiratory or neurologic signs of unknown etiology.

## Figures and Tables

**Table 1:** Epidemiological and laboratory data of patients with confirmed or probable fatal flea-borne typhus in Texas, 1985–2015

Patient (age/sex)	Ethnicity	Animal exposure	Flea exposure	Onset (month/year)	Days from onset to death	Case status	Reciprocal IgG IFA titer(s) to *Rickettsia typhi*
81/F	Hispanic	Yes*†	Unknown	June 1985	11	Confirmed	64, 1,024
67/M	Non-Hispanic	No	No	September 1986	17	Probable	256
36/M	Hispanic	Unknown	Unknown	June 1991	8	Confirmed	≥1,024
72/M	Hispanic	Unknown	Unknown	June 1995	34	Confirmed	≥1,024
73/F	Hispanic	Yes†	Yes	December 1998	6	Probable	512§
53/M	Hispanic	Unknown	Unknown	January 2007	6	Confirmed	≥1,024
36/M	Non-Hispanic	Yes*‡	Yes	April 2012	13	Confirmed	≥4,096
50/M	Non-Hispanic	Yes†	Unknown	May 2013	20	Confirmed	≥4,096¶
55/M	Hispanic	Yes†‡	Yes	May 2013	14	Probable	256
84/F	Hispanic	Unknown	Unknown	November 2014	12	Probable	64∥
62/M	Unknown	Yes‡	Unknown	January 2015	9	Probable	256**

IFA = indirect immunofluorescence antibody.

Reported exposure to: * opossum, † cat, ‡ dog.

§ Patient also had 1:40 titer to *R. typhi* by slide agglutination assay.

¶ Patient also had a skin biopsy specimen positive for a typhus group *Rickettsia* sp.by immunohistochemical stain.

∥ Patient also had a reciprocal IgM IFA titer of 512 on same date.

** Patient also had a reciprocal IgM IFA titer of 2,048 on same date.

**Table 2:** Clinical characteristics of 11 patients with fatal flea-borne typhus in Texas, 1985–2015

Patient (age/sex)	Fever	Thrombocytopenia	Elevated hepatic transaminases	Anorexia	Nausea/vomiting	Rash	Pneumonia	Headache	Coma	Cough	Encephalopathy	Pulmonary edema	Acute kidney injury	Meningitis	Vertigo	Acute respiratory distress syndrome
81/F*†	√				√		√						√			
67/M	√					√	√		√					√		
36/M‡	√	√			√											
72/M*	√	√	√	√	√			√								
73/F*‡	√	√	√	√		√										
53/M‡	√	√	√	√	√	√			√							
36/M‡	√	√	√	√	√			√		√						
50/M*‡	√	√				√			√							
55/M*‡	√	√	√	√		√		√		√	√				√	
84/F*†‡	√	√	√	√	√		√				√	√				
62/M*‡	√	√	√			√						√	√			√
Total (%)	11 (100)	9 (82)	7 (63)	6 (55)	6 (55)	6 (55)	3 (27)	3 (27)	3 (27)	2 (18)	2 (18)	2 (18)	2 (18)	1 (9)	1 (9)	1 (9)

* Patients that received tetracycline-class antibiotic during hospitalization.

† Patients that received sulfa drug before hospitalization.

‡ Medical charts available for review.
